# Characteristics Associated With Out-of-Hospital Cardiac Arrests and Resuscitations During the Novel Coronavirus Disease 2019 Pandemic in New York City

**DOI:** 10.1001/jamacardio.2020.2488

**Published:** 2020-06-19

**Authors:** Pamela H. Lai, Elizabeth A. Lancet, Michael D. Weiden, Mayris P. Webber, Rachel Zeig-Owens, Charles B. Hall, David J. Prezant

**Affiliations:** 1 Office of Medical Affairs, Fire Department of the City of New York, Brooklyn, New York; 2 Bureau of Health Services, Fire Department of the City of New York, Brooklyn, New York; 3 Pulmonary, Critical Care and Sleep Medicine Division, Department of Medicine and Department of Environmental Medicine, New York University School of Medicine, New York; 4 Division of Epidemiology, Department of Epidemiology and Population Health, Albert Einstein College of Medicine, Bronx, New York; 5 Pulmonary Medicine Division, Department of Medicine, Montefiore Medical Center and Albert Einstein College of Medicine, Bronx, New York; 6 Division of Biostatistics, Department of Epidemiology and Population Health, Albert Einstein College of Medicine, Bronx, New York

## Abstract

**Importance:**

Risk factors for out-of-hospital death due to novel coronavirus disease 2019 (COVID-19) are poorly defined. From March 1 to April 25, 2020, New York City, New York (NYC), reported 17 118 COVID-19–related deaths. On April 6, 2020, out-of-hospital cardiac arrests peaked at 305 cases, nearly a 10-fold increase from the prior year.

**Objective:**

To describe the characteristics (race/ethnicity, comorbidities, and emergency medical services [EMS] response) associated with outpatient cardiac arrests and death during the COVID-19 pandemic in NYC.

**Design, Setting, and Participants:**

This population-based, cross-sectional study compared patients with out-of-hospital cardiac arrest receiving resuscitation by the NYC 911 EMS system from March 1 to April 25, 2020, compared with March 1 to April 25, 2019. The NYC 911 EMS system serves more than 8.4 million people.

**Exposures:**

The COVID-19 pandemic.

**Main Outcomes and Measures:**

Characteristics associated with out-of-hospital arrests and the outcomes of out-of-hospital cardiac arrests.

**Results:**

A total of 5325 patients were included in the main analysis (2935 men [56.2%]; mean [SD] age, 71 [18] years), 3989 in the COVID-19 period and 1336 in the comparison period. The incidence of nontraumatic out-of-hospital cardiac arrests in those who underwent EMS resuscitation in 2020 was 3 times the incidence in 2019 (47.5/100 000 vs 15.9/100 000). Patients with out-of-hospital cardiac arrest during 2020 were older (mean [SD] age, 72 [18] vs 68 [19] years), less likely to be white (611 of 2992 [20.4%] vs 382 of 1161 [32.9%]), and more likely to have hypertension (2134 of 3989 [53.5%] vs 611 of 1336 [45.7%]), diabetes (1424 of 3989 [35.7%] vs 348 of 1336 [26.0%]), and physical limitations (2259 of 3989 [56.6%] vs 634 of 1336 [47.5%]). Compared with 2019, the odds of asystole increased in the COVID-19 period (odds ratio [OR], 3.50; 95% CI, 2.53-4.84; *P* < .001), as did the odds of pulseless electrical activity (OR, 1.99; 95% CI, 1.31-3.02; *P* = .001). Compared with 2019, the COVID-19 period had substantial reductions in return of spontaneous circulation (ROSC) (727 of 3989 patients [18.2%] vs 463 of 1336 patients [34.7%], *P* < .001) and sustained ROSC (423 of 3989 patients [10.6%] vs 337 of 1336 patients [25.2%], *P* < .001), with fatality rates exceeding 90%. These associations remained statistically significant after adjustment for potential confounders (OR for ROSC, 0.59 [95% CI, 0.50-0.70; *P* < .001]; OR for sustained ROSC, 0.53 [95% CI, 0.43-0.64; *P* < .001]).

**Conclusions and Relevance:**

In this population-based, cross-sectional study, out-of-hospital cardiac arrests and deaths during the COVID-19 pandemic significantly increased compared with the same period the previous year and were associated with older age, nonwhite race/ethnicity, hypertension, diabetes, physical limitations, and nonshockable presenting rhythms. Identifying patients with the greatest risk for out-of-hospital cardiac arrest and death during the COVID-19 pandemic should allow for early, targeted interventions in the outpatient setting that could lead to reductions in out-of-hospital deaths.

## Key Points

**Question:**

What characteristics are associated with out-of-hospital cardiac arrests and death during the COVID-19 pandemic in New York City?

**Findings:**

In this population-based cross-sectional study of 5325 patients with out-of-hospital cardiac arrests, the number undergoing resuscitation was 3-fold higher during the 2020 COVID-19 period compared with during the comparison period in 2019. Patients with out-of-hospital cardiac arrest during 2020 were older, less likely to be white, and more likely to have specific comorbidities and substantial reductions in return and sustained return of spontaneous circulation.

**Meaning:**

Identifying patients at risk for out-of-hospital cardiac arrest and death during the COVID-19 pandemic should lead to interventions in the outpatient setting to help reduce out-of-hospital deaths.

## Teaser

This cross-sectional study describes the characteristics associated with outpatient cardiac arrests and death during the coronavirus disease 2019 pandemic in New York City.

## Introduction

On March 1, 2020, the first case of novel coronavirus disease 2019 (COVID-19) was diagnosed in New York City, New York (NYC); by April 25, 2020, 17 118 confirmed and probable deaths due to COVID-19 had already occurred.^1^ On April 6, 2020, NYC out-of-hospital cardiac arrests peaked at 305 cases, an increase of almost 10-fold compared with April 6, 2019. In Northern Italy, during the COVID-19 pandemic, out-of-hospital cardiac arrests increased by 58% compared with the same time period in 2019 and were associated with lower rates of sustained return of spontaneous circulation (ROSC).^2^ Infectious viral epidemics causing severe respiratory infections have long been associated with an increased risk of death.^3,4,5,6,7^ For the COVID-19 pandemic, factors independently associated with in-hospital deaths included being older than 65 years, hypertension, diabetes, cardiovascular disease, and chronic obstructive pulmonary disease (COPD).^8^

To date, factors associated with out-of-hospital cardiac arrests and successful resuscitation during the COVID-19 pandemic have not been defined. Using data from the NYC 911 emergency medical services (EMS) system, our study compared patients with nontraumatic out-of-hospital cardiac arrest who received resuscitation during the COVID-19 period and their outcomes with patients and outcomes during the same period in 2019. Our goal was to identify COVID-19–associated changes in frequency, risk factors, presenting cardiac rhythm, and out-of-hospital death despite EMS resuscitation.

## Methods

This study followed the Strengthening the Reporting of Observational Studies in Epidemiology (STROBE) reporting guideline. The institutional review board of the Montefiore Medical Center, Albert Einstein College of Medicine, Bronx, New York, approved this study and, owing to minimal risk to the participants (ie, no effect on their rights and welfare), waived the need for informed consent.

### Data Sources

The NYC 911 EMS system is the largest in the United States, serving a population of more than 8.4 million and responding to more than 1.5 million medical calls annually. This 3-tiered system consists of firefighter-certified first responders, emergency medical technician basic life support units, and paramedic advanced life support (ALS) units. In the NYC 911 system, cardiac arrests receive the highest response priority and all 3 units (firefighter-certified first responders, basic life support units, and ALS units) are immediately dispatched. Both firefighter-certified first responders and basic life support units are certified in basic cardiac life support and carry automated external defibrillators. Paramedic ALS units can obtain and interpret 12-lead electrocardiograms and are certified in advanced cardiac life support, including advanced airway management and administering cardiac resuscitation medications. Out-of-hospital cardiac arrests are managed by EMS responders using regional prehospital protocols modeled after the American Heart Association Guidelines for Cardiopulmonary Resuscitation and Emergency Cardiovascular Care.^9^

Data on out-of-hospital cardiac arrests are collected and managed by the Fire Department of NYC Online Medical Control. For cases in which EMS resuscitation is performed, a postresuscitation telephone interview of paramedics and emergency medical technicians is conducted by Online Medical Control staff. The questionnaire collects data in the Utstein style^10^ on age, sex, race/ethnicity, preexisting comorbidities, bystander cardiopulmonary resuscitation (CPR), presenting rhythm, and advanced cardiac life support interventions (airway management and medications). The questionnaire was validated in prior cardiac arrest research within our system.^11^ Interviews are supplemented with information from electronic prehospital patient care reports completed by EMS responders. Final call-type of cardiac arrest, response time, and ALS first on-scene were obtained from the Fire Department of NYC’s 911 computer automated dispatch system. All data are maintained in a secure data warehouse.

### Study Design

This population-based, cross-sectional study included patients 18 years or older with out-of-hospital cardiac arrest who received EMS resuscitation during the COVID-19 period (March 1 to April 25, 2020) or the comparison period (March 1 to April 25, 2019) in NYC. The COVID-19 period was chosen to begin March 1, 2020, the date the first patient was diagnosed with COVID-19 in NYC, and to conclude on April 25, 2020, when EMS call volume approached its pre–COVID-19 baseline. The comparison period was chosen to mirror the COVID-19 dates during the previous year. Patients with out-of-hospital cardiac arrests were excluded if they did not undergo prehospital CPR owing to obvious signs of death or had a valid do-not-resuscitate order present at the time of arrest (n = 3601). The COVID-19 and the 2019 periods had similar proportions of patients dead on arrival (2355 [35.1%] vs 831 [36.1%], respectively) and patients with a do-not-resuscitate order (323 [4.8%] vs 92 [4.0%], respectively). The number of traumatic arrests were similar in the 2 periods (42 vs 43, respectively). Our final population with confirmed, nontraumatic cardiac arrest resuscitations accounted for 60% of total confirmed cardiac arrests during the study period.

### Data Analysis

First, we examined characteristics (demographic and other) of individuals with confirmed, nontraumatic out-of-hospital cardiac arrests who underwent resuscitation during the 2 study periods. The assumption was that excess cases of out-of-hospital cardiac arrests in the COVID-19 period were likely associated with the COVID-19 pandemic, either directly or indirectly. Excess cases of out-of-hospital cardiac arrest resuscitations were calculated by taking the daily difference between the number of calls in 2020 and 2019. The cumulative percentage of EMS calls for fever, cough, dyspnea, and viral-like symptoms consistent with COVID-19 and the cumulative percentage of excess out-of-hospital cardiac arrest resuscitations were calculated, and the temporal relationship graphed. Second, we compared the association of COVID-19 with out-of-hospital ROSC and ROSC that was sustained until emergency department arrival (hereinafter referred to as sustained ROSC), adjusted for known covariates of ROSC and sustained ROSC. These covariates included age (in 10-year increments), race/ethnicity, sex, medical history, EMS response time, bystander CPR, ALS first on-scene, ALS interventions, and presenting rhythm. For the models, first-unit response time was recoded from a continuous time variable to a binary variable of less than 6 minutes (yes or no). A response time of less than 6 minutes has been shown in multiple studies and national registries^12^ to provide the most benefit to out-of-hospital cardiac arrest outcomes.^5,13^ Last, presenting rhythm was only captured for cases in which ALS personnel were the first arriving units, because basic life support units or firefighter-certified first responders cannot confirm presenting rhythm when first on the scene. Because this reduced the sample available for our multivariate models, the variable was recoded to include an unclassified category to encompass cases in which presenting rhythm was missing.

### Statistical Analysis

Unadjusted outcomes were compared using descriptive statistics. Categorical data were compared using Pearson χ^2^, whereas continuous data were compared using 2-tailed *t* tests or medians and interquartile ranges. Multivariable logistic regression analyses were performed to identify characteristics of patients with out-of-hospital cardiac arrest in the COVID-19 period as well as to assess the association of the COVID-19 period with ROSC and sustained ROSC, controlling for the above covariates. A 2-sided *P* < .05 was considered statistically significant for both unadjusted and adjusted analyses.

Two sensitivity analyses using these same outcomes were conducted. The first compared the peak 2-week period of out-of-hospital cardiac arrests during the COVID-19 period (March 29 to April 11, 2020) with the same 2-week period in 2019. The second compared the peak 2-week COVID-19 period with that of the 2 weeks just before (March 16-28, 2020) and after (April 12-25, 2020) that peak. Analyses were conducted in SAS, version 9.4 (SAS Institute Inc).

## Results

A total of 5325 patients were included in the main analysis (2935 men [56.2%] and 2292 women [43.9%]; mean [SD] age, 71 [18] years). Compared with 2019, 2020 had an excess of 2653 patients with out-of-hospital cardiac arrest who underwent EMS resuscitation (3989 in 2020 vs 1336 in 2019, *P* < .001), an incidence rate triple that of 2019 (47.5/100 000 vs 15.9/100 000). No time lag was observed between the proportion of daily NYC 911 EMS calls for fever, cough, dyspnea, and viral-like symptoms consistent with COVID-19 and excess out-of-hospital cardiac arrest resuscitations, defined as the difference between 2020 and 2019 counts each day (Figure, A). Figure, B shows the number of resuscitations in the population, as described herein, by period.

**Figure.**
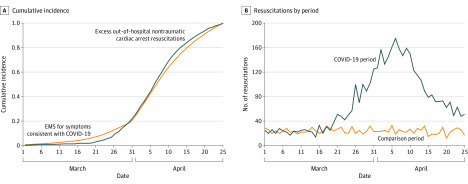
New York City Out-of-Hospital Nontraumatic Cardiac Arrest Resuscitations, March 1 through April 25, 2020 A, Temporal association between the cumulative percentage of emergency medical services (EMS) calls for fever, cough, dyspnea, and viral-like symptoms consistent with coronavirus disease 2019 (COVID-19) and the number of excess out-of-hospital nontraumatic cardiac arrest resuscitations occurring in New York City in 2020. Excess cases were defined as the daily difference between the number of 2020 and 2019 cases; days with a negative difference were recoded as 0 for graphic presentation. B, The number of daily out-of-hospital nontraumatic cardiac arrest resuscitations.

Table 1 displays the characteristics of patients with out-of-hospital nontraumatic cardiac arrests who underwent EMS resuscitation during each period. The patients with out-of-hospital cardiac arrest in 2020 were older (mean [SD] age, 72 [18] vs 68 [19] years); less likely to be white (611 of 2992 [20.4%] vs 382 of 1161 [32.9%]); and more likely to have hypertension (2134 of 3989 [53.5%] vs 611 of 1336 [45.7%]), diabetes (1424 of 3989 [35.7%] vs 348 of 1336 [26.0%]), and physical limitations (2259 of 3989 [56.6%] vs 634 of 1336 [47.5%]). Alternately, 2020 patients with out-of-hospital cardiac arrest did not have higher proportions of prior cardiac disease, asthma/COPD, cerebrovascular accidents, or cancer. The proportions of bystander-witnessed arrests and bystander CPR were similar in both periods.

**Table 1.** Patient Characteristics of Out-of-Hospital Nontraumatic Cardiac Arrests During COVID-19 and 1 Year Before^a^

Characteristic	Cardiac arrest resuscitations			
	Main analysis (n = 5325)^b^		Sensitivity analysis peak period (n = 2292)^c^	
	Comparison period (n = 1336)	COVID-19 period (n = 3989)	Comparison period (n = 341)	COVID-19 period (n = 1951)
Age, mean (SD), y	68 (19)	72 (18)	69 (18)	72 (15)
Male^d^	752 (57.1)	2183 (55.8)	200 (59.9)	1085 (57.0)
Race^e^				
White	382 (32.9)	611 (20.4)	87 (30.3)	244 (17.6)
Asian	88 (7.6)	218 (7.3)	19 (6.6)	96 (6.9)
Black	332 (28.6)	1025 (34.3)	89 (31.0)	486 (35.0)
Hispanic	239 (20.6)	763 (25.5)	64 (22.3)	391 (28.1)
Mixed	120 (10.3)	375 (12.5)	28 (9.8)	172 (12.4)
Medical history				
Cardiac disease	397 (29.7)	1008 (25.3)	105 (30.8)	465 (23.8)
Hypertension	611 (45.7)	2134 (53.5)	157 (46.0)	1039 (53.3)
Diabetes	348 (26.0)	1424 (35.7)	81 (23.8)	708 (36.3)
Renal disease	105 (7.9)	313 (7.8)	28 (8.2)	137 (7.0)
Asthma/COPD	214 (16.0)	509 (12.8)	57 (16.7)	227 (11.6)
Cancer	125 (9.4)	282 (7.1)	39 (11.4)	114 (5.8)
CVA	90 (6.7)	228 (5.7)	20 (5.9)	114 (5.8)
Physical limitations	634 (47.5)	2259 (56.6)	164 (48.1)	1128 (57.8)
Bystander witnessed	404 (30.2)	1080 (27.1)	106 (31.1)	516 (26.4)
Bystander CPR	441 (33.0)	1359 (34.1)	109 (32.0)	657 (33.7)
Presenting rhythm^f^				
Ventricular rhythms^g^	38 (11.0)	45 (3.6)	13 (13.8)	17 (2.9)
Asystole	209 (60.6)	973 (77.6)	53 (56.4)	475 (80.7)
PEA	72 (20.9)	177 (14.1)	21 (22.3)	64 (10.9)

Abbreviations: COPD, chronic obstructive pulmonary disease; COVID-19, coronavirus disease 2019; CPR, cardiopulmonary resuscitation; CVA, cerebrovascular accident; PEA, pulseless electrical activity.

^a^ Study population used for these estimates includes only patients who received resuscitation by emergency medical services. Unless otherwise indicated, data are expressed as number (percentage) of patients.

^b^ Covers March 1 to April 25, 2019 (comparison period) and 2020 (COVID-19 period).

^c^ Covers March 29 to April 11, 2019 (comparison period) and 2020 (COVID-19 period).

^d^ Owing to missing data, includes 1316 for the main analysis comparison period, 3915 for the main analysis COVID-19 period, 334 for the sensitivity analysis comparison period, and 1902 for the sensitivity analysis COVID-19 period.

^e^ Owing to missing data, includes 1161 for the main analysis comparison period, 2992 for the main analysis COVID-19 period, 287 for the sensitivity analysis comparison period, and 1389 for the sensitivity analysis COVID-19 period.

^f^ Owing to missing data, includes 345 for the main analysis comparison period, 1254 for the main analysis COVID-19 period, 94 for the sensitivity analysis comparison period, and 589 for the sensitivity analysis COVID-19 period. Presenting rhythm data were only collected for those out-of-hospital cardiac arrests in which an advanced life support unit was first on the scene.

^g^ Ventricular rhythms include ventricular fibrillation and ventricular tachycardia.

In our multivariate model of patient characteristics, we found that compared with 2019, out-of-hospital cardiac arrest resuscitations during the COVID-19 period were associated with increasing age (odds ratio [OR], 1.12; 95% CI, 1.07-1.18; *P* < .001), nonwhite race/ethnicity (eg, OR for Hispanic, 2.06 [95% CI, 1.68-2.52; *P* < .001]; OR for black, 1.90 [95% CI, 1.57-2.29; *P* < .001]), a history of diabetes (OR, 1.45; 95% CI, 1.23-1.71; *P* < .001) or hypertension (OR, 1.28; 95% CI, 1.09-1.50; *P* = .002), and physical limitations (OR, 1.27; 95% CI, 1.09-1.49; *P* = .002) (Table 2). By contrast, the odds of cardiac disease, asthma/COPD, cancer, and cerebrovascular accidents were not increased in 2020 relative to 2019. During the COVID-19 period, out-of-hospital cardiac arrests were 3.5 times more likely to present in asystole (OR, 3.50; 95% CI, 2.53-4.84; *P* < .001) and twice as likely to present in pulseless electrical activity (OR, 1.99; 95% CI, 1.31-3.02; *P* = .001) than in ventricular rhythms (ventricular fibrillation or ventricular tachycardia).

**Table 2.** Association of Risk Factors With Out-of-Hospital Nontraumatic Cardiac Arrests in the COVID-19 Period vs 1 Year Before^a^

Risk factor	Main analysis^b^		Sensitivity analysis peak period^c^	
	OR (95% CI)	*P* value	OR (95% CI)	*P* value
Age (per 10 y)	1.12 (1.07-1.18)	<.001	1.16 (1.05-1.27)	.003
Sex				
Female	1 [Reference]		1 [Reference]	
Male	0.92 (0.79-1.06)	.25	1.05 (0.79-1.39)	.74
Race/ethnicity				
White	1 [Reference]		1 [Reference]	
Asian	1.43 (1.08-1.91)	.01	1.82 (1.01-3.28)	.05
Black	1.90 (1.57-2.29)	<.001	1.91 (1.33-2.74)	<.001
Hispanic	2.06 (1.68-2.52)	<.001	2.28 (1.54-3.37)	<.001
Mixed	1.96 (1.52-2.53)	<.001	1.99 (1.21-3.28)	.007
Medical history				
Cardiac disease	0.72 (0.61-0.86)	<.001	0.67 (0.49-0.93)	.02
Hypertension	1.28 (1.09-1.50)	.002	1.27 (0.94-1.73)	.12
Diabetes	1.45 (1.23-1.71)	<.001	1.81 (1.31-2.51)	<.001
Renal disease	0.79 (0.60-1.03)	.08	0.64 (0.39-1.06)	.09
Asthma/COPD	0.78 (0.64-0.95)	.02	0.67 (0.45-0.99)	.05
Cancer	0.72 (0.56-0.92)	.009	0.59 (0.37-0.94)	.03
CVA	0.70 (0.52-0.94)	.02	0.74 (0.41-1.32)	.30
Physical limitations	1.27 (1.09-1.49)	.002	1.38 (1.03-1.86)	.04
Presenting rhythm^d^				
Ventricular rhythms^e^	1 [Reference]		1 [Reference]	
Asystole	3.50 (2.53-4.84)	<.001	5.37 (3.01-9.58)	<.001
PEA	1.99 (1.31-3.02)	.001	2.77 (1.29-5.91)	.009

Abbreviations: COPD, chronic obstructive pulmonary disease; COVID-19, coronavirus disease 2019; CVA, cerebrovascular accident; OR, odds ratio; PEA, pulseless electrical activity.

^a^ Study population used for these estimates includes only those out-of-hospital cardiac arrests who received resuscitation by emergency medical services.

^b^ Covers March 1 to April 25, 2019 (comparison period) and 2020 (COVID-19 period).

^c^ Covers March 29 to April 11, 2019 (comparison period) and 2020 (COVID-19 period).

^d^ Presenting rhythm data were only collected for those out-of-hospital cardiac arrests in which an advanced life support unit was first on the scene; however, an unclassified category was added for missing data to bolster model observations.

^e^ Ventricular rhythms include ventricular fibrillation and ventricular tachycardia.

Table 3 displays ALS interventions and outcomes for patients with out-of-hospital cardiac arrest who underwent EMS resuscitation during each period. Rates of ROSC (727 of 3989 patients [18.2%] vs 463 of 1336 patients [34.7%]; *P* < .001) and sustained ROSC (423 of 3989 patients [10.6%] vs 337 of 1336 patients [25.2%]; *P* < .001) were significantly lower during the COVID-19 period than in 2019. Furthermore, patients during the COVID-19 period were significantly more likely to have resuscitation terminated in the field compared with patients from the 2019 period (3566 of 3989 [89.4%] vs 999 of 1336 [74.8%]; *P* < .001), reflecting the inability to obtain ROSC or sustained ROSC after at least 20 minutes of resuscitation. When examining only excess cases, resuscitation terminated in 2020 increased to 2567 of 2653 patients (96.8%). Despite marked differences in outcome, bystander-witnessed arrests, bystander CPR, time to first unit on the scene, time to ALS on the scene, and duration of resuscitation were similar in both time periods.

**Table 3.** Outcomes of Patients With Out-of-Hospital Nontraumatic Cardiac Arrest Resuscitations in the COVID-19 Period vs 1 Year Before^a^

Variable	Cardiac arrest resuscitations			
	Main analysis (n = 5325)^b^		Sensitivity analysis peak period (n = 2292)^c^	
	Comparison period (n = 1336)	COVID-19 period (n = 3989)	Comparison period (n = 341)	COVID-19 period (n = 1951)
ROSC, No. (%)^d^	463 (34.7)	727 (18.2)	133 (39.0)	297 (15.2)
Sustained ROSC, No. (%)	337 (25.2)	423 (10.6)	96 (28.2)	153 (7.8)
Resuscitation terminated in field	999 (74.8)	3566 (89.4)	245 (71.9)	1798 (92.2)
Time to first unit on scene, median (IQR), min:s	5:05 (2:17-7:13)	5:56 (2:14-9:38)	5:03 (2:36-7:30)	6:38 (2:08-10:28)
Time to ALS unit on scene, median (IQR), min:s	7:32 (2:27-13:17)	9:60 (0:43-19:17)	7:22 (2:24-12:20)	11:17 (1:07-22:07)
Total resuscitation time, median (IQR), min:s	34:58 (20:15-49:01)	32:18 (16:33-48:03)	35:17 (21:14-49:20)	30:55 (16:18-45:32)
ALS unit first on scene, No. (%)	345 (25.8)	1254 (31.4)	94 (27.6)	589 (30.2)
Shock delivered prior to ALS unit arrival, No. (%)	79 (5.9)	109 (2.7)	20 (5.9)	43 (2.2)
Airway, No. (%)				
Endotracheal intubation	1011 (75.7)	1915 (48.0)	245 (71.8)	825 (42.3)
Supraglottic airway	193 (14.4)	1385 (34.7)	59 (17.3)	706 (36.2)
Bag valve mask	132 (9.9)	689 (17.3)	37 (10.9)	420 (21.5)
Medications administered, No. (%)				
None	75 (5.6)	455 (11.4)	27 (7.9)	312 (16.0)
Epinephrine	1238 (92.7)	3516 (88.1)	310 (90.9)	1633 (83.7)
Amiodarone	143 (10.7)	231 (5.8)	33 (9.7)	77 (3.9)
Dextrose	193 (14.4)	328 (8.2)	40 (11.7)	132 (6.8)
Sodium bicarbonate	598 (44.8)	909 (22.8)	164 (48.1)	302 (15.5)
Naloxone	89 (6.7)	67 (1.7)	19 (5.6)	17 (0.9)
Magnesium	43 (3.2)	38 (1.0)	4 (1.2)	9 (0.5)

Abbreviations: ALS, advanced life support; COVID-19, coronavirus disease 2019; IQR, interquartile range; ROSC, return of spontaneous circulation.

^a^ Study population used for these estimates includes only those with out-of-hospital cardiac arrests who received resuscitation by emergency medical services.

^b^ Covers March 1 to April 25, 2019 (comparison period) and 2020 (COVID-19 period).

^c^ Covers March 29 to April 11, 2019 (comparison period) and 2020 (COVID-19 period).

^d^ ROSC and sustained ROSC are not mutually exclusive, but patients must be either in sustained ROSC or have resuscitation terminated.

To assess for potential confounding, multivariate logistic regression (Table 4) confirmed that compared with 2019, patients with out-of-hospital cardiac arrest in 2020 were 41% less likely to attain ROSC (OR, 0.59; 95% CI, 0.50-0.70; *P* < .001) and 47% less likely to attain sustained ROSC (OR, 0.53; 95% CI, 0.43-0.64; *P* < .001). Additional risk factors for failure to achieve ROSC or sustained ROSC include female sex, black race/ethnicity, ALS not being the first unit on the scene, receiving airway management other than endotracheal intubation, and receiving no advanced cardiac life support medications. Response time of at least 6 minutes was associated with a lower likelihood of sustained ROSC (OR, 1.38; 95% CI, 1.13-1.69; *P* = .002). Compared with ventricular rhythms, presenting rhythms of asystole and pulseless electrical activity were significantly associated with lower likelihood of achieving ROSC (OR for asystole, 0.26 [95% CI, 0.17-0.41; *P* < .001]; OR for pulseless electrical activity, 0.56 [95% CI, 0.33-0.95; *P* = .03]) or sustained ROSC (OR for asystole, 0.25 [95% CI, 0.15-0.41; *P* < .001]; OR for pulseless electrical activity, 0.50 [95% CI, 0.29-0.89; *P* = .02]).

**Table 4.** Association of COVID-19 With ROSC and Sustained ROSC Adjusted for Demographics, Interventions, and Response Time^a^

Intervention or risk factor	ROSC				Sustained ROSC			
	Main analysis		Sensitivity analysis peak period		Main analysis		Sensitivity analysis peak period	
	OR (95% CI)	*P* value^b^	OR (95% CI)	*P* value^b^	OR (95% CI)	*P* value^b^	OR (95% CI)	*P* value^b^
Study period								
Comparison	1 [Reference]		1 [Reference]		1 [Reference]		1 [Reference]	
COVID-19	0.59 (0.50-0.70)	<.001	0.44 (0.32-0.60)	<.001	0.53 (0.43-0.64)	<.001	0.38 (0.26-0.56)	<.001
Age (per 10 y)	1.00 (0.96-1.05)	.97	0.92 (0.84-1.01)	.07	0.97 (0.91-1.02)	.25	0.90 (0.81-1.01)	.07
Sex								
Female	1 [Reference]		1 [Reference]		1 [Reference]		1 [Reference]	
Male	1.53 (1.31-1.79)	<.001	1.55 (1.18-2.04)	.002	1.59 (1.32-1.93)	<.001	1.60 (1.13-2.25)	.007
Race/ethnicity								
White	1 [Reference]		1 [Reference]		1 [Reference]		1 [Reference]	
Asian	1.11 (0.81-1.52)	.52	1.09 (0.62-1.91)	.76	1.16 (0.80-1.68)	.44	0.77 (0.37-1.61)	.49
Black	0.79 (0.64-0.98)	.03	0.73 (0.50-1.06)	.10	0.68 (0.53-0.87)	.002	0.60 (0.38-0.95)	.03
Hispanic	0.89 (0.72-1.12)	.33	0.94 (0.64-1.39)	.77	0.86 (0.66-1.11)	.25	0.78 (0.48-1.25)	.30
Mixed	1.12 (0.85-1.48)	.41	1.10 (0.67-1.80)	.70	1.21 (0.89-1.65)	.23	1.19 (0.66-2.12)	.56
Medical history								
Cardiac disease	1.13 (0.94-1.36)	.18	0.93 (0.67-1.28)	.65	1.30 (1.05-1.61)	.02	1.16 (0.78-1.72)	.47
Hypertension	0.98 (0.83-1.17)	.84	1.09 (0.81-1.46)	.59	1.00 (0.82-1.23)	.98	1.12 (0.77-1.63)	.56
Diabetes	0.93 (0.77-1.11)	.39	0.78 (0.58-1.06)	.11	0.85 (0.68-1.05)	.14	0.83 (0.56-1.23)	.35
Renal disease	1.22 (0.92-1.62)	.17	1.29 (0.79-2.12)	.31	1.28 (0.92-1.79)	.15	0.95 (0.49-1.82)	.87
Asthma/COPD	1.30 (1.05-1.61)	.02	1.20 (0.82-1.76)	.35	1.32 (1.03-1.70)	.03	1.27 (0.80-2.03)	.31
Cancer	1.11 (0.83-1.47)	.48	0.98 (0.60-1.63)	.95	0.98 (0.70-1.39)	.93	0.81 (0.42-1.56)	.52
CVA	0.75 (0.52-1.06)	.11	1.01 (0.56-1.81)	.97	0.83 (0.55-1.25)	.36	1.33 (0.67-2.63)	.41
Physical limitations	0.56 (0.47-0.67)	<.001	0.74 (0.55-1.00)	.05	0.59 (0.47-0.73)	<.001	0.73 (0.50-1.07)	.10
Bystander CPR								
No	1 [Reference]		1 [Reference]		1 [Reference]		1 [Reference]	
Yes	1.18 (0.99-1.40)	.06	1.27 (0.95-1.71)	.11	1.31 (1.07-1.60)	.009	1.52 (1.06-2.19)	.02
Response time, min								
≥6	1 [Reference]		1 [Reference]		1 [Reference]		1 [Reference]	
<6	1.09 (0.93-1.28)	.30	1.06 (0.81-1.40)	.66	1.38 (1.13-1.69)	.002	1.32 (0.93-1.88)	.12
ALS unit first on scene								
No	1 [Reference]		1 [Reference]		1 [Reference]		1 [Reference]	
Yes	2.44 (1.60-3.73)	<.001	2.70 (1.31-5.58)	.007	2.84 (1.81-4.45)	<.001	3.29 (1.49-7.22)	.003
ALS medication administration								
No	1 [Reference]		1 [Reference]		1 [Reference]		1 [Reference]	
Yes	1.65 (1.08-2.52)	.02	2.46 (1.24-4.89)	.01	1.25 (0.78-1.99)	.36	1.81 (0.80-4.07)	.15
Airway maintenance								
Endotracheal intubation	1 [Reference]		1 [Reference]		1 [Reference]		1 [Reference]	
Supraglottic airway	0.48 (0.40-0.58)	<.001	0.52 (0.39-0.71)	<.001	0.41 (0.32-0.52)	<.001	0.50 (0.33-0.74)	<.001
Bag valve mask	0.50 (0.37-0.68)	<.001	0.50 (0.29-0.85)	.01	0.60 (0.42-0.86)	.005	0.57 (0.29-1.09)	.09
Presenting rhythm^c^								
Ventricular rhythms^d^	1 [Reference]		1 [Reference]		1 [Reference]		1 [Reference]	
Asystole	0.26 (0.17-0.41)	<.001	0.21 (0.10-0.44)	<.001	0.25 (0.15-0.41)	<.001	0.16 (0.07-0.36)	<.001
PEA	0.56 (0.33-0.95)	.03	0.60 (0.25-1.47)	.27	0.50 (0.29-0.89)	.02	0.36 (0.13-0.98)	.05

Abbreviations: ALS, advanced life support; COPD, chronic obstructive pulmonary disease; COVID-19, coronavirus disease 2019; CPR, cardiopulmonary resuscitation; CVAs, cerebrovascular accidents; NA, not applicable; OR, odds ratio; PEA, pulseless electrical activity; ROSC, return of spontaneous circulation.

^a^ Study population used for these estimates includes only those with out-of-hospital cardiac arrests who received resuscitation by emergency medical services. Main study period covered March 1 to April 25, 2019 (comparison period) and 2020 (COVID-19 period); sensitivity time period, March 29 to April 11, 2019 (comparison period) and 2020 (COVID-19 period).

^b^ Calculated using logistic regression.

^c^ Presenting rhythm data were only collected for those out-of-hospital cardiac arrests in which an ALS unit was first on the scene; however, an unclassified category was added for missing data to bolster model observations.

^d^ Ventricular rhythm includes ventricular fibrillation and ventricular tachycardia.

Our 2 sensitivity analyses revealed the same associations as did our main model (Table 2). For ROSC and sustained ROSC, results from our sensitivity analysis were nearly identical to those in Table 4 in the direction of associations, although some factors lost statistical significance for ROSC (being black and having a presenting rhythm of pulseless electrical activity) or for sustained ROSC (history of asthma/COPD, physical activity limitations, and a response time of ≥6 minutes). Return of spontaneous circulation and sustained ROSC were both significantly lower during the 2-week COVID-19 peak period in both sensitivity analyses. For example, when compared with the same 2 weeks in 2019, ROSC occurred in 297 of 1951 patients with out-of-hospital nontraumatic cardiac arrest (15.2%) vs 133 of 341 patients (39.0%) (*P* < .001) and sustained ROSC was attained in 153 of 1951 patients (7.8%) vs 96 of 341 patients (28.2%) (*P* < .001). Similarly, when compared with the 2 weeks before plus the 2 weeks after the peak 2020 period, ROSC occurred in 297 of 1951 patients (15.2%) vs 430 of 2038 patients (21.1%) (*P* < .001), and sustained ROSC occurred in 153 of 1951 patients (7.8%) vs 270 of 2038 patients (13.3%) (*P* < .001).

## Discussion

Using data from the NYC 911 EMS system during the COVID-19 pandemic, we report 2653 excess out-of-hospital cardiac arrests, a number that, by itself, represents double the number of patients with out-of-hospital cardiac arrests who underwent EMS resuscitation during the comparable 2019 period. More than 90% of these excess cases resulted in out-of-hospital deaths, some of which likely contributed to the 17 118 confirmed and suspected COVID-19–related deaths that occurred in NYC during the first 8 weeks of the pandemic. Risk factors for excess COVID-19–related out-of-hospital cardiac arrests included older age and minority race/ethnicity, after adjustment for comorbidities. Importantly, nonshockable presenting rhythms of asystole and pulseless electrical activity were more commonly documented in 2020 compared with 2019 and likely account for the substantial increase in out-of-hospital cardiac arrest mortality.

Conditions associated with COVID-19, including hypoxemic respiratory failure, massive myocardial infarction, and pulmonary emboli, can lead to rapid decompensation and result in cardiac arrest with initial nonshockable rhythms.^14,15,16^ Our results were similar to those observed in Northern Italy, where out-of-hospital cardiac arrests increased by 58% from the same time period in 2019.^2^ Italy had an increase in out-of-hospital mortality from 67.3% to 82.2% and an increase of initial nonshockable rhythms from 83% to 90%.^2^ In Wuhan, China, unsuccessful resuscitation for in-hospital cardiac arrests occurred 86.8% of the time, with 89.7% of patients having asystole as the initial presenting rhythm.^17^

Increased out-of-hospital cardiac arrests during influenza are thought to be due to the body’s systemic inflammatory response, which destabilizes atherosclerotic plaques that, in turn, produce myocardial infarctions and cardiovascular deaths.^5,7,18^ In addition to overwhelming pneumonia, viral sepsis, and acute respiratory failure,^19^ COVID-19 causes endothelial injury predisposing to thrombosis in the arterial and venous system with myocardial infarction in the absence of atherosclerosis and increased risk of venous thromboembolism.^20,21,22,23^ Declining oxygenation and biomarkers of tissue injury (elevated levels of cardiac troponins, cytokines, D-dimer, and lactate) are risk factors for death in hospitalized patients with COVID-19.^24,25^

Similar to risk factors for death in hospitalized patients, we found that increasing age, hypertension, and diabetes were independent risk factors for patients with out-of-hospital cardiac arrest during 2020. We also observed that patients reported to have physical activity limitations, such as being bed or wheelchair bound, were at increased risk for COVID-19–related out-of-hospital cardiac arrests. Immobility may be a marker for frailty and is a risk factor for thromboembolic disease. Although sex, asthma/COPD, prior cardiac disease, and cerebrovascular accidents are known risk factors for in-hospital cardiac deaths,^26,27,28,29^ they were not risk factors in our study of excess out-of-hospital cardiac arrests in 2020. This may be because these comorbidities were risks for out-of-hospital cardiac arrests^30^ in the comparison 2019 period and therefore did not contribute significantly to excess out-of-hospital cardiac arrest cases in 2020.

In our study, minority race/ethnicity was a risk factor for COVID-19–related out-of-hospital cardiac arrests even after adjusting for comorbidities that disproportionately affect minority populations. Black, Hispanic, and Asian patients were at increased risk for COVID-19–associated out-of-hospital cardiac arrests and death. Explanations for these disparities are multifactorial, difficult to disaggregate, and range from individual vulnerabilities to social/environmental factors. The disparate burden of out-of-hospital cardiac arrests in minority populations may be a consequence of underlying comorbidities, genetic-environmental interactions, socioeconomic conditions that include increased viral exposure due to crowding and reduced opportunity to work from home, as well as reduced access to health care.^31^

Although we observed a temporal association (without time lag) between NYC 911 EMS calls for fever, cough, dyspnea, and viral-like symptoms and out-of-hospital cardiac arrests, that in itself is insufficient to demonstrate that excess out-of-hospital cardiac arrests and deaths after attempted resuscitation were solely owing to sudden cardiopulmonary decompensation from COVID-19 infection. The observed temporal relationship does not preclude other explanations, such as the possibility that delays in seeking or receiving health care may have negatively affected slowly progressive COVID-19 infections or preexisting conditions (eg, cardiopulmonary diseases or cancer), resulting in out-of-hospital cardiac arrests and deaths. During this period, hospitals reported few admissions for other conditions,^32^ and in Italy, admission rates for acute coronary syndrome significantly declined.^33^ Reasons for such delays may include not only lack of health care access but also purposeful avoidance due to fears of contracting COVID-19. In addition, pandemic-related environmental, emotional, and economic stressors could have indirectly contributed to excess out-of-hospital cardiac arrests and deaths. Because our data cannot address the proportion of out-of-hospital cardiac arrests that was directly or indirectly due to COVID-19, further research is needed. Even before the results of further research are available, the increased COVID-19–related out-of-hospital cardiac arrest rates in our study reinforce the need for improved health care outreach during pandemics, especially for vulnerable populations.

Our results agree with established findings of higher rates of sustained ROSC with shorter EMS response time.^12,13,30^ With the increased number of patients presenting with COVID-19–like symptoms, the median response time of available EMS units to out-of-hospital cardiac arrests was increased by approximately 1 minute; however, this difference was not statistically significant when compared with the same period in 2019. Although the time range was variable, the median response time was less than the 3-minute increase reported in Italy.^2^ In contrast, if ALS units arrived first on the scene, we observed significantly higher rates of ROSC and sustained ROSC compared with other units, even during the COVID-19 period. Studies characterizing the association of prehospital ALS management with out-of-hospital cardiac arrest in the pre–COVID-19 era report conflicting results.^34,35,36,37^ In our study, ALS interventions (ACLS medications and endotracheal intubation) were associated with significant increases in both ROSC and sustained ROSC (Table 4) in all analyses. We speculate that these ALS interventions were more likely to occur and to be successful when ALS units were first on the scene. In addition, paramedics’ higher training and medical knowledge provide critical skills in patient assessment that lead to effective treatment decisions and team-based leadership^35^ during resuscitations.

During the COVID-19 study period, less invasive airway management (supraglottic airway or bag-valve-mask ventilation) was associated with lower rates of ROSC and sustained ROSC. Several studies, including a meta-analysis, have shown increased ROSC rates and overall survival to hospital discharge with endotracheal intubation,^38,39^ although the mechanism for this improvement has not yet been elucidated. The significant decrease in the use of more invasive procedures, such as endotracheal intubation, in favor of less invasive procedures (supraglottic airways and bag-valve-mask ventilation) may be due to EMS responders wanting to reduce exposure to the patient during the COVID-19 pandemic. This may have been a concern despite the availability of personal protective equipment, including fresh N95 masks, eye protection, gowns, and gloves that were supplied to and required of all personnel during resuscitations. This finding was similarly observed in the management of out-of-hospital cardiac arrests in Italy at the height of their response to COVID-19.^2^

### Limitations

Our study shares several limitations found in recently published COVID-19 in-hospital mortality studies. First, our study population was limited to those who received care, in this case, EMS resuscitation. Second, because postmortem testing to confirm COVID-19 was rarely performed, we cannot distinguish between increased cardiopulmonary arrests directly due to COVID-19 or indirectly due to unattended comorbid diseases during this pandemic. Support for the increase in out-of-hospital cardiac arrests being directly COVID-19 related in our study and for a similar trend in the study from Italy is based on comparisons with the prior year. We acknowledge that although cardiovascular disease, asthma/COPD, cerebrovascular accidents, and cancer were not risk factors for out-of-hospital cardiac arrests during the COVID-19 pandemic in our study, patient lack of access to or avoidance of health care leading to acute decompensation of comorbid illnesses may have played a role. We do not believe that reliance on prehospital patient information for comorbid history resulted in differential misclassification, because the same method was used in both periods (2019 and 2020), and the percentage of bystander-witnessed events was similar. Ultimately, corroboration by death certificates, along with autopsy studies, is required to determine the proportion of out-of-hospital cardiac arrests and deaths that were related to COVID-19. A strength of the current investigation is the longitudinal, system-wide ascertainment of out-of-hospital cardiac arrests and resuscitations in the largest US 911 system during the largest pandemic since the 1918 influenza pandemic.^40^ By including data from the entire 911 system and comparing it with the same time period 1 year prior, the potential for differential ascertainment biases was minimized. By choosing the longer period as our main analysis rather than the 2-week COVID-19 peak period, we purposely biased our results toward the null.

## Conclusions

The tragedy of the COVID-19 pandemic is not just the number of patients infected, but the large increase in out-of-hospital cardiac arrests and deaths. This catastrophe transpired despite similar rates of bystander CPR, similar EMS response times, and similar durations of resuscitation efforts, compared with 2019. The findings of this cross-sectional study emphasize the importance of intervening early in the course of COVID-19 infection, before acute decompensation. They also speak to the critical need to design better systems for providing health care access to vulnerable, at-risk patients with acute and chronic conditions during a pandemic. Aggressive efforts for identifying outpatient risk factors for out-of-hospital cardiac arrests and death, such as hypoxia and hypercoagulability, especially in minority populations, should be instituted. Further research is needed to determine if early, targeted interventions in the outpatient setting for those at risk, such as regular telemedicine visits and home-based monitoring of vital signs, oxygen saturation, and biomarkers of tissue injury in those that test positive could lead to reductions in out-of-hospital fatalities.

## References

[1] NYC Department of Health and Mental Hygiene COVID-19: data: coronavirus-data/case-hosp.death.csv. 2020. Accessed May 7, 2020 https://github.com/nychealth/coronavirus-data

[2] Baldi E, Sechi GM, Mare C, ; Lombardia CARe Researchers Out-of-hospital cardiac arrest during the Covid-19 outbreak in Italy. N Engl J Med. 2020. doi:10.1056/NEJMc2010418 32348640 PMC7204428

[3] Thompson WW, Shay DK, Weintraub E, Mortality associated with influenza and respiratory syncytial virus in the United States. JAMA. 2003;289(2):179-186. doi:10.1001/jama.289.2.179 12517228

[4] Thompson WW, Shay DK, Weintraub E, Influenza-associated hospitalizations in the United States. JAMA. 2004;292(11):1333-1340. doi:10.1001/jama.292.11.1333 15367555

[5] Nguyen JL, Yang W, Ito K, Matte TD, Shaman J, Kinney PL Seasonal influenza infections and cardiovascular disease mortality. JAMA Cardiol. 2016;1(3):274-281. doi:10.1001/jamacardio.2016.0433 27438105 PMC5158013

[6] Madjid M, Miller CC, Zarubaev VV, Influenza epidemics and acute respiratory disease activity are associated with a surge in autopsy-confirmed coronary heart disease death: results from 8 years of autopsies in 34,892 subjects. Eur Heart J. 2007;28(10):1205-1210. doi:10.1093/eurheartj/ehm035 17440221 PMC7108465

[7] Madjid M, Aboshady I, Awan I, Litovsky S, Casscells SW Influenza and cardiovascular disease: is there a causal relationship? Tex Heart Inst J. 2004;31(1):4-13. 15061620 PMC387426

[8] CDC COVID-19 Response Team Severe outcomes among patients with coronavirus disease 2019 (COVID-19): United States, February 12-March 16, 2020. MMWR Morb Mortal Wkly Rep. 2020;69(12):343-346. doi:10.15585/mmwr.mm6912e2 32214079 PMC7725513

[9] Link MS, Berkow LC, Kudenchuk PJ, Part 7: adult advanced cardiovascular life support: 2015 American Heart Association guidelines update for cardiopulmonary resuscitation and emergency cardiovascular care. Circulation. 2015;132(18)(suppl 2):S444-S464. doi:10.1161/CIR.0000000000000261 26472995

[10] Jacobs I, Nadkarni V, Bahr J, ; International Liason Committee on Resusitation Cardiac arrest and cardiopulmonary resuscitation outcome reports: update and simplification of the Utstein templates for resuscitation registries: a statement for healthcare professionals from a task force of the International Liaison Committee on Resuscitation (American Heart Association, European Resuscitation Council, Australian Resuscitation Council, New Zealand Resuscitation Council, Heart and Stroke Foundation of Canada, InterAmerican Heart Foundation, Resuscitation Council of Southern Africa). Resuscitation. 2004;63(3):233-249. doi:10.1016/j.resuscitation.2004.09.008 15582757

[11] Freese J, Hall CB, Lancet EA, Intra-arrest induction of hypothermia via large-volume ice-cold saline for sudden cardiac arrest: the New York City Project Hypothermia Experience. Ther Hypothermia Temp Manag. 2019;9(2):128-135. doi:10.1089/ther.2018.0023 30427769

[12] Cardiac Arrest Registry to Enhance Survival 2018 Annual report. Accessed May 1, 2020. https://mycares.net/sitepages/uploads/2019/2018_flipbook/index.html?page=1

[13] Vukmir RB Survival from prehospital cardiac arrest is critically dependent upon response time. Resuscitation. 2006;69(2):229-234. doi:10.1016/j.resuscitation.2005.08.014 16500015

[14] Carr GE, Yuen TC, McConville JF, ; American Heart Association’s Get With the Guidelines-Resuscitation (National Registry of CPR) Investigators Early cardiac arrest in patients hospitalized with pneumonia: a report from the American Heart Association’s Get With The Guidelines-Resuscitation Program. Chest. 2012;141(6):1528-1536. doi:10.1378/chest.11-1547 22194592 PMC3367483

[15] Kürkciyan I, Meron G, Sterz F, Pulmonary embolism as a cause of cardiac arrest: presentation and outcome. Arch Intern Med. 2000;160(10):1529-1535. doi:10.1001/archinte.160.10.1529 10826469

[16] Virkkunen I, Paasio L, Ryynänen S, Pulseless electrical activity and unsuccessful out-of-hospital resuscitation: what is the cause of death? Resuscitation. 2008;77(2):207-210. doi:10.1016/j.resuscitation.2007.12.006 18249482

[17] Shao F, Xu S, Ma X, In-hospital cardiac arrest outcomes among patients with COVID-19 pneumonia in Wuhan, China. Resuscitation. 2020;151:18-23. doi:10.1016/j.resuscitation.2020.04.005 32283117 PMC7151543

[18] Takano T, Tajiri H, Kashiwagi Y, Kimura S, Kawashima H Cytokine and chemokine response in children with the 2009 pandemic influenza A (H1N1) virus infection. Eur J Clin Microbiol Infect Dis. 2011;30(1):117-120. doi:10.1007/s10096-010-1041-9 20820834 PMC2998638

[19] Richardson S, Hirsch JS, Narasimhan M, ; Northwell COVID-19 Research Consortium Presenting characteristics, comorbidities, and outcomes among 5700 patients hospitalized with COVID-19 in the New York City area. JAMA. 2020;323(20):2052-2059. doi:10.1001/jama.2020.6775 32320003 PMC7177629

[20] Magro C, Mulvey JJ, Berlin D, Complement associated microvascular injury and thrombosis in the pathogenesis of severe COVID-19 infection: a report of five cases. Transl Res. Published online April 15, 2020. doi:10.1016/j.trsl.2020.04.007 32299776 PMC7158248

[21] Mehra MR, Desai SS, Kuy S, Henry TD, Patel AN Cardiovascular disease, drug therapy, and mortality in Covid-19. N Engl J Med. 2020. doi:10.1056/NEJMoa2007621 32356626 PMC7206931

[22] Klok FA, Kruip M, van der Meer NJM, Incidence of thrombotic complications in critically ill ICU patients with COVID-19. Thromb Res. 2020;191:145-147. 10.1016/j.thromres.2020.04.013 PMC7146714 32291094

[23] Guzik TJ, Mohiddin SA, Dimarco A, COVID-19 and the cardiovascular system: implications for risk assessment, diagnosis, and treatment options. Cardiovasc Res. Published online April 30, 2020. doi:10.1093/cvr/cvaa106 32352535 PMC7197627

[24] Spiezia L, Boscolo A, Poletto F, COVID-19-related severe hypercoagulability in patients admitted to intensive care unit for acute respiratory failure. Thromb Haemost. Published online April 21, 2020. doi:10.1055/s-0040-1710018 32316063 PMC7295272

[25] Connors JM, Levy JH Thromboinflammation and the hypercoagulability of COVID-19. J Thromb Haemost. 2020. doi:10.1111/jth.14849 32302453 PMC9770920

[26] Epidemiology Working Group for NCIP Epidemic Response, Chinese Center for Disease Control and Prevention. The epidemiological characteristics of an outbreak of 2019 novel coronavirus diseases (COVID-19) in China [in Chinese]. Zhonghua Liu Xing Bing Xue Za Zhi. 2020;41(2):145-151. 10.3760/cma.j.issn.0254-6450.2020.02.003 32064853

[27] Garg S, Kim L, Whitaker M, Hospitalization rates and characteristics of patients hospitalized with laboratory-confirmed coronavirus disease 2019: COVID-NET, 14 states, March 1-30, 2020. MMWR Morb Mortal Wkly Rep. 2020;69(15):458-464. doi:10.15585/mmwr.mm6915e3 32298251 PMC7755063

[28] Zhou F, Yu T, Du R, Clinical course and risk factors for mortality of adult inpatients with COVID-19 in Wuhan, China: a retrospective cohort study. Lancet. 2020;395(10229):1054-1062. doi:10.1016/S0140-6736(20)30566-3 32171076 PMC7270627

[29] Ruan Q, Yang K, Wang W, Jiang L, Song J Clinical predictors of mortality due to COVID-19 based on an analysis of data of 150 patients from Wuhan, China. Intensive Care Med. 2020;46(5):846-848. doi:10.1007/s00134-020-05991-x PMC7080116 32125452

[30] Virani SS, Alonso A, Benjamin EJ, ; American Heart Association Council on Epidemiology and Prevention Statistics Committee and Stroke Statistics Subcommittee Heart disease and stroke statistics—2020 update: a report from the American Heart Association. Circulation. 2020;141(9):e139-e596. doi:10.1161/CIR.0000000000000757 31992061

[31] Pareek M, Bangash MN, Pareek N, Ethnicity and COVID-19: an urgent public health research priority. Lancet. 2020;395(10234):1421-1422. doi:10.1016/S0140-6736(20)30922-3 32330427 PMC7173801

[32] Baum A, Schwartz MD Admissions to Veterans Affairs hospitals for emergency conditions during the COVID-19 pandemic. JAMA. Published online June 5, 2020. doi:10.1001/jama.2020.9972 32501493 PMC7275263

[33] De Filippo O, D’Ascenzo F, Angelini F, Reduced rate of hospital admissions for ACS during Covid-19 outbreak in Northern Italy. N Engl J Med. 2020. doi:10.1056/NEJMc2009166 32343497 PMC7224608

[34] Stiell IG, Wells GA, Field B, ; Ontario Prehospital Advanced Life Support Study Group Advanced cardiac life support in out-of-hospital cardiac arrest. N Engl J Med. 2004;351(7):647-656. doi:10.1056/NEJMoa040325 15306666

[35] Wang HE, Kupas DF Outcomes after out-of-hospital cardiac arrest treated by basic vs advanced life support. JAMA Intern Med. 2015;175(8):1421. doi:10.1001/jamainternmed.2015.2097 26236974

[36] Sanghavi P, Jena AB, Newhouse JP, Zaslavsky AM Outcomes after out-of-hospital cardiac arrest treated by basic vs advanced life support. JAMA Intern Med. 2015;175(2):196-204. doi:10.1001/jamainternmed.2014.5420 25419698 PMC4314335

[37] Kurz MC, Schmicker RH, Leroux B, ; ROC Investigators Advanced vs basic life support in the treatment of out-of-hospital cardiopulmonary arrest in the Resuscitation Outcomes Consortium. Resuscitation. 2018;128:132-137. doi:10.1016/j.resuscitation.2018.04.031 29723609

[38] Benoit JL, Gerecht RB, Steuerwald MT, McMullan JT Endotracheal intubation versus supraglottic airway placement in out-of-hospital cardiac arrest: a meta-analysis. Resuscitation. 2015;93:20-26. doi:10.1016/j.resuscitation.2015.05.007 26006743

[39] McMullan J, Gerecht R, Bonomo J, ; CARES Surveillance Group Airway management and out-of-hospital cardiac arrest outcome in the CARES registry. Resuscitation. 2014;85(5):617-622. doi:10.1016/j.resuscitation.2014.02.007 24561079

[40] Olson DR, Simonsen L, Edelson PJ, Morse SS Epidemiological evidence of an early wave of the 1918 influenza pandemic in New York City. Proc Natl Acad Sci U S A. 2005;102(31):11059-11063. doi:10.1073/pnas.0408290102 16046546 PMC1182402

